# Compassionate paediatric anaesthesia: still a long way to go. Comment on *BJA Open* 2025; 14: 100411

**DOI:** 10.1016/j.bjao.2026.100858

**Published:** 2026-09-07

**Authors:** Kristin Keunen, Piet L. Leroy

**Affiliations:** 1 Department of Anaesthesiology, Amsterdam University Medical Centres, Amsterdam, The Netherlands; 2 Department of Paediatrics, School of Health Professions Education, Maastricht University Medical Centre, Maastricht University, Maastricht, The Netherlands

**Keywords:** induction of anaesthesia, paediatric anaesthesia, parental presence, preoperative anxiety

Preoperative anxiety in children remains a significant challenge for anaesthesiologists. Matava and colleagues^1^ provided important insight into its incidence across multiple children's hospitals in the USA, Canada, and the UK, retrospectively reviewing 155 604 paediatric encounters. Using the Child Induction Behavioural Assessment tool^2^ and Mask Acceptance Scale,^3^ the authors found significant anxiety behaviours in 22.2% of children, difficult induction in 6.2%, and difficult mask acceptance in 20% of patients. All incidences peaked in children aged 1–3 yr (40.8%, 11.5%, and 34.2%, respectively). Difficult induction incidence was lower with the use of premedication. Notably, parental presence at induction was associated with higher odds of difficult induction. The authors concluded that their study provides valuable real-world insights into preoperative anxiety and underscores the need for individually tailored, patient-centred care.

In their eloquent editorial, Hansen and colleagues^4^ highlighted critical elements for consideration of the elaborate work by Matava and colleagues.^1^ We share their view that this study has substantial methodological limitations that may hinder the interpretation of its findings. Without explicit practical guidance, we believe that the findings of Matava and colleagues risk being misinterpreted as an argument against parental presence at induction and/or favouring premedication as the preferred method of choice for anxiety reduction. We caution against premature conclusions and would like to add key elements to the dialogue that we believe are critical to address.

Before determining which strategy most effectively manages a child's preoperative anxiety, we must first establish professional consensus on what good care encompasses. Although medical, operational, and logistical considerations are unequivocally relevant, we believe that a child's fundamental right to good care should serve as the primary guiding principle. Hansen and colleagues^4^ highlighted children's rights to receive emotional support during stressful medical procedures in general and to parental presence in particular. Recently, extensive rights-based standards for healthcare professionals have been published that specifically address children's rights during medical procedures such as anaesthesia induction.^5^ The following standard is specifically relevant for this discussion: the right to ‘be cared for by professionals who have the appropriate knowledge and skills to support a child's physical, emotional and psychological well-being and rights before, during and after their procedure’. It follows that professionals caring for children should be competent not only in safe and effective medical care but also in safeguarding a child's emotional and psychological safety. Parental presence is a fundamental component of a child's emotional safety and is most critical in infants and toddlers (0–3 yr), whose neurological immaturity precludes effective self-regulation during distress.^6^, ^7^, ^8^ This concept is corroborated by results of a systematic review and meta-analysis collating data from more than 700 children, demonstrating that parental presence during induction reduces both child and parental anxiety.^9^ Furthermore, it is associated with reduced operating room times.

Several studies highlighted that parental presence involves more than mere physical proximity and is most effective when parents are calm, confident, and well prepared for their role.^10^, ^11^, ^12^ Therefore, anaesthesia providers bear some responsibility for optimising parental preparation and engagement. Both mask practice and parental distraction in the preoperative holding area have been identified as effective preparation strategies for mask induction.^10^ Similar exposure and desensitisation practices may be helpful in i.v. anaesthesia induction. Signs of parental anxiety and distress—sometimes more subtle than in children—should be actively sought and addressed through clear pre-procedural explanation while offering parents a defined, actively engaging, and comforting role.^13^^,^^14^ Importantly, parents should never be involved in restraining their child.

Not all evidence on parental presence is unequivocally positive.^15^ Matava and colleagues^1^ reported that parental presence was associated with higher odds of difficult anaesthesia induction. The retrospective and quantitative design of their study precludes a detailed understanding of what parental presence entailed.^1^ Moreover, no data were reported on anaesthesia providers' attitudes towards, and expertise with, parental presence. This is a crucial omission given that providers who routinely practise parental presence are more likely to appreciate its benefits.^9^ Without these insights, it is difficult to draw meaningful conclusions from the observations.

Second, we would like to stress that premedication should be used with caution. When used to *support* the child (and their parent/caregiver) in undergoing the procedure comfortably and fluently, premedication may be a key element within a broader approach aimed at establishing genuine comfort and trust. However, critically, it should never replace non-pharmacological comfort strategies, nor should it be used merely to facilitate the logistical and procedural processes. Studies showed that midazolam—often used as premedication—interferes with explicit memory formation while preserving implicit memory.^16^^,^^17^ Therefore, children may unconsciously remember the potentially stressful event of anaesthesia induction, but may not consciously be able to make sense of the experience. Consequently, traumatic memory building cannot be excluded. Consistent with this concern, Dutch paediatric anaesthesia guidelines advocate that, when premedication is used, non-pharmacological comfort strategies (e.g. parental active presence, calm environmental conditions) should be adopted simultaneously.^18^ Matava and colleagues^1^ found that premedication was administered in 33.4% of children but was combined with other comfort strategies in only 5.7% of patients. Thus, it was used as a sole intervention in 27.7% of inductions. This practice suggests substantial room for improvement.

Although debate remains on which premedication agent is optimal in children, meta-analyses reveal that alpha_2_-adrenergic agonists are most effective as a single agent.^19^^,^^20^ Notably, a systematic review and meta-analysis including 123 adult trials found that benzodiazepines have no effect on recovery quality or postoperative pain in adults and may increase postoperative anxiety.^21^ These findings were echoed in a recent paediatric randomised controlled trial showing superior outcomes with alpha_2_-adrenergic agonists compared with midazolam, including less pain and a lower incidence of delirium and postoperative anxiety.^22^ Among non-pharmacological interventions, both passive distraction (e.g. listening to music, watching a video) and interactive distraction (e.g. playing video games, playing with toys), combined with parental presence, demonstrate the greatest benefit in reducing anxiety during anaesthesia induction in children.^23^

The most important contribution of the work by Matava and colleagues^1^ is demonstrating that preoperative anxiety in paediatric anaesthesia remains alarmingly prevalent and is likely underestimated by current assessment tools. The study's large, real-world dataset is a considerable strength. However, the study results stop short of translating these findings into actionable clinical recommendations. Nonetheless, as anaesthesia providers, we are well positioned to drive meaningful improvement in patient care through proactive parental engagement, family-centred preparation, thoughtful integration of non-pharmacological comfort measures, and the judicious use of premedication. We advocate for a tailored approach that incorporates past experiences and takes the child's and parents' preferences into account and is therefore best planned during preoperative screening visits. Support from child life specialists or premedication can be involved if indicated. Parental presence, preferably with active engagement, should be the blueprint of any strategy.

## Authors’ contributions

Writing—original draft, reviewing, and editing: KK

Writing—original draft, reviewing, and editing: PLL

## Declarations of interest

The authors declare that they have no conflicts of interest.
